# Caught in the Act: Live-Cell Imaging of Plant Meiosis

**DOI:** 10.3389/fpls.2021.718346

**Published:** 2021-12-21

**Authors:** Maria Ada Prusicki, Martina Balboni, Kostika Sofroni, Yuki Hamamura, Arp Schnittger

**Affiliations:** Department of Developmental Biology, Institute for Plant Science and Microbiology, University of Hamburg, Hamburg, Germany

**Keywords:** chromosome, recombination, microscopy, cell division, culture media, fluorescent reporter, time course

## Abstract

Live-cell imaging is a powerful method to obtain insights into cellular processes, particularly with respect to their dynamics. This is especially true for meiosis, where chromosomes and other cellular components such as the cytoskeleton follow an elaborate choreography over a relatively short period of time. Making these dynamics visible expands understanding of the regulation of meiosis and its underlying molecular forces. However, the analysis of meiosis by live-cell imaging is challenging; specifically in plants, a temporally resolved understanding of chromosome segregation and recombination events is lacking. Recent advances in live-cell imaging now allow the analysis of meiotic events in plants in real time. These new microscopy methods rely on the generation of reporter lines for meiotic regulators and on the establishment of *ex vivo* culture and imaging conditions, which stabilize the specimen and keep it alive for several hours or even days. In this review, we combine an overview of the technical aspects of live-cell imaging in plants with a summary of outstanding questions that can now be addressed to promote live-cell imaging in Arabidopsis and other plant species and stimulate ideas on the topics that can be addressed in the context of plant meiotic recombination.

## Introduction

Meiosis is a specialized cell division process required for sexual reproduction. It consists of one round of DNA replication followed by two consecutive events of chromosome segregation that result in four genetically different cells with half the DNA content of the mother cell (e.g., haploid meiotic products are formed in diploid organisms). In animals, meiosis directly produces the gametes. By contrast, the meiotic products of plants, called spores, undergo several cell divisions, from just a few in vascular plants such as Arabidopsis (*Arabidopsis thaliana*) and maize (*Zea mays*) to many in non-vascular plants such as the moss *Physcomitrium* (*Physcomitrella*) *patens* and the genus *Marchantia*, to produce a gametophyte. The mature gametophyte harbors the actual gametes. In the case of flowering plants, including Arabidopsis and maize, the female gametophyte holds an egg cell and a central cell embedded in the embryo sac; the male gametophyte contains two sperm cells encapsulated in a pollen grain (Hater et al., 2020; Hafidh and Honys, 2021).

Some of the first observations of meiosis, dating from the late 19th century, were made by Oscar Hertwig, who studied sea urchins, and Eduard Van Beneden, who investigated the nematode *Ascaris megalocephala* (Hertwig, 1876; Van Beneden, 1883). Since then, microscopy has become a vital approach to investigate meiosis. In particular, cell spreads, immunostaining, and fluorescence *in situ* hybridization (FISH) analyses have been used to assemble the current extensive knowledge of meiosis (Keeney, 2009; Pradillo and Heckmann, 2020). These methods offer excellent spatial resolution, especially when subjected to super-resolution microscopy such as structured illumination microscopy (SIM), stimulated emission depletion (STED), and stochastic optical reconstruction microscopy (STORM), which have revealed the organization of the cohesin and synaptonemal complex (SC) in, for example, fruit fly (*Drosophila melanogaster*), the nematode *Caenorhabditis elegans*, mouse (*Mus musculus*), and recently Arabidopsis (Cahoon et al., 2017; Hernandez et al., 2018; Yoon et al., 2018; Xu et al., 2019; Sims et al., 2021).

However, as these techniques rely on fixed meiocytes, they give only a snapshot of the dynamic events taking place during the two cell divisions. Specifically, it is difficult to analyze meiotic progression in a heterogeneous population in which some cells behave differently from others: e.g., proceeding through meiosis at different paces and adopting different cellular configurations. Moreover, chromosome spreading procedures inherently rely on disrupting a higher-order three-dimensional structure and collapsing it onto a two-dimensional surface to make local chromatin details visible, such as the co-localization pattern of the recombinases DISRUPTED MEIOTIC cDNA1 (DMC1) and RADIATION51 (RAD51) (Reitz et al., 2019). In addition, the washing steps of immunolocalization experiments can also affect the pattern and abundance of biological structures and molecules, especially when they localize to the cytoplasm or nucleoplasm. On one hand, these washing steps can help enhance or reveal a specific localization pattern; for instance, the association of CYCLIN-DEPENDENT KINASE A;1 (CDKA;1) with chromatin in male meiotic cells in Arabidopsis only became visible after the nucleoplasmic fraction of CDKA;1 was reduced by the washing steps during immunolocalization experiments (Bulankova et al., 2010; Zhao et al., 2012; Yang et al., 2020). On the other hand, by changing the relative distribution of antigens, immunolocalization data deliver only a limited perspective of the situation found in nature. For instance, the above-mentioned nucleoplasmic localization of CDKA;1 might in fact be biologically relevant. Moreover, not all epitopes are always accessible to an antibody, further decreasing the levels or aspects of the detected proteins.

Recent advances in microscopy, such as the improved light-gathering and detection sensitivity of laser scanning and spinning disk confocal microscope systems and the development of (lattice) light sheet microscopy, have made it possible to obtain cytological data in three and even four dimensions and to follow the course of meiosis in real time with little perturbation.

Early live-cell imaging studies of meiosis were conducted in fission yeast (*Schizosaccharomyces pombe*), budding yeast (*Saccharomyces cerevisiae*), and Drosophila in the 1990s (Chikashige et al., 1994; Smith et al., 1995; Matthies et al., 1996). The work in fission and budding yeasts was based on wide-field and fluorescence microscopy. Chikashige et al. (1994) monitored nuclear movements in fission yeast, attributing a leading function to the telomeres during the horse-tail configuration, which is a specific prophase stage characterized by parallel chromosome threads that extend longitudinally from one side of the nucleus to the other. Matthies et al. (1996) used a laser confocal scanning microscope (LCSM) to follow the dynamics of spindle assembly in Drosophila oocytes, which revealed that meiotic spindle formation in this organism does not depend on the presence of microtubule organizing centers, but rather is organized by the chromosomes.

Key questions in meiotic research regarding the mechanism, function, and regulation of chromosome pairing, telomere bouquet formation, CO formation, and spindle formation have since then been assessed by live-cell imaging, which has provided new insights. These studies included further analyses in fission yeast (Tomita and Cooper, 2007), budding yeast (Conrad et al., 2008; Koszul and Kleckner, 2009; Lee et al., 2012, 2020), and Drosophila (Hughes et al., 2011; Colombié et al., 2013; Christophorou et al., 2015), as well as *C. elegans* (Vargas et al., 2019), and mammalian cells (Schuh and Ellenberg, 2007; Kitajima et al., 2011; Holubcová et al., 2013; Lee et al., 2015; Pfender et al., 2015; Kyogoku and Kitajima, 2017; Mogessie and Schuh, 2017; Enguita-Marruedo et al., 2018; Nikalayevich et al., 2018; Silva et al., 2020; Wang et al., 2020).

An extensive discussion of the use of live-cell imaging of animal and yeast meiosis goes beyond the scope of this review. Therefore, we only highlight a few examples here that opened new research directions in meiosis and should stimulate equivalent lines of research in plants, as illustrated by the work of Kyogoku and Kitajima (2017) and Wang et al. (2020), in which the authors studied the biophysical regulation of meiosis, which is normally not accessible in fixed material. To this end, Kyogoku and Kitajima combined micromanipulation of cell size and cell shape with live-cell imaging. This work revealed that the large size of the oocyte correlates with errors in chromosome bi-orientation and with a less stringent spindle assembly checkpoint due to a low nucleus-to-cytoplasm ratio and therefore to the frequent aneuploidy of mammal oocytes (Kyogoku and Kitajima, 2017). Wang et al. (2020) succeeded in measuring the cytoplasmic stream and the underlying hydrodynamic forces generated, which correlated with the correct extrusion of the second polar body after meiosis II in mammalian oocytes.

Finally, live-cell imaging has been instrumental in performing new genetic screens (Pfender et al., 2015). Pfender et al. (2015) studied the function of 774 genes involved in meiosis using small interfering RNA (siRNA)–mediated silencing coupled with live-cell imaging. Groups of 12 siRNAs were injected into early-stage oocytes, which were still embedded into follicles, to induce knock-down phenotypes. Once the oocytes were fully grown, they were extruded and injected with mRNAs for *GFP-a-tubulin* (a-tubulin fused to the green fluorescent protein [GFP]) and *H2B-mRFP* (histone H2B fused to the red fluorescent protein [RFP]) and incubated for 2–3 h to allow the translation of the fluorescent proteins before observation on a LCSM. Multiple cells were imaged in parallel, in four dimensions, as previously described (Schuh and Ellenberg, 2007). A manual evaluation of the phenotypes led to the description of 50 meiotic disturbances, including presence of lagging chromosomes, spindle length, or absence of nuclear envelope breakdown (NEB), which each corresponded to malfunctions of single genes, including previously unknown genes.

## Live-Cell Imaging of Meiosis in Plants: Technical Aspects

**FIGURE 1:**
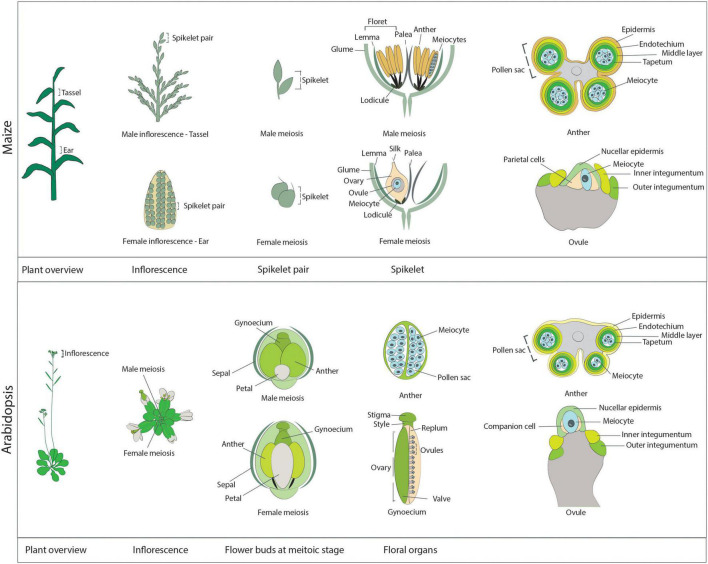
Schematic overview of the reproductive structures harboring meiocytes in maize **(upper panel)** and Arabidopsis **(lower panel)**. Maize: A maize plant at the meiotic stage. The immature male inflorescence, the tassel, is located at the last internode, while the immature female inflorescence, the ear, is positioned at the base of leaves in the midsection of the plant. The individual reproductive units of each inflorescence are the spikelets, which occur in pairs. Each spikelet comprises two florets, subtended by a pair of glumes. On the tassel, each floret contains a lemma, a palea, and three anthers, which harbor the male meiocytes. The maize anther at the meiotic stage is approximately 4 mm in length. As seen from the transverse section of an anther (right-most diagram), the meiocytes occupy the inner part of each of the four pollen sacs forming the anther and are surrounded by four cell layers: the tapetum, the middle layer, the endothecium, and the epidermis. A meiotic ear is approximately 20 mm in length. In each ear, only one of the two florets is functional, while the other floret degenerates. Each functional floret harbors the ovary, which contains one ovule enclosing the meiocyte and the parietal cells. The ovule structure at the meiotic stage is characterized by the presence of the inner and outer integuments, which elongate on each side of the nucellus. Arabidopsis: An Arabidopsis plant at the meiotic stage. Each inflorescence consists of multiple flower buds at different developmental stages. Each flower bud contains the male floral organs (six anthers) and the female floral organ (the gynoecium). These reproductive organs are surrounded by four petals and four sepals. Male meiosis takes place in flower buds when they are approximately 0.8 mm in length, are round in shape (not elongated), and contain very small petals that do not cover the anthers, which are approximately 0.2 mm in length. The transverse section of an Arabidopsis anther reveals a structure similar to that of maize anthers: The meiocytes occupy the inner part of each of the four pollen sacs, surrounded by the tapetum, the middle layer, the endothecium, and the epidermis. Female meiosis takes place in elongated flower buds that are approximately 1.2 mm in length (hence slightly later than male meiosis; at this stage anthers are elongated and start to get a yellow shade). The gynoecium, or pistil, reaches approximately 0.9 mm in length at the meiotic stage. It is composed of the stigma, the style, and the ovary, which contains multiple ovules, connected to the replum and protected by valves. As with maize, at the meiotic stage, primordia of the inner and outer integuments are visible rising on the side of the nucellus, while inside the nucellus it is possible to identify a single meiocyte and a pair of companion cells.

In contrast to animal and yeast systems, live-cell imaging in plants has not been a prominent technique to study meiosis in the past, with only a few articles presenting live-cell imaging data until recently (Yu et al., 1997; Sheehan and Pawlowski, 2009; Higgins et al., 2016; Nannas et al., 2016; Ingouff et al., 2017). This fact is surprising for several reasons. First, live-cell imaging is extensively used to analyze various aspects of plant development and physiology, e.g., plant reproduction and the sensing of metabolites (Higashiyama et al., 2001; Okuda et al., 2009; Jones et al., 2014). Second, due to their large chromosomes and, hence, exquisite cell biology, many plant species (for instance, lily [*Lilium* sp.] and maize) are often used as model systems to study cell division, reaching as far back as one of the first optical description of mitosis by Strasburger (1888). One possible explanation for the lag in applying live-cell imaging to plant meiosis may stem from the challenges associated with directly observing plant meiocytes, as they are buried deep within reproductive tissues (Figure 1).

Recently, complementary experimental setups have been developed to overcome these shortcomings for the imaging of plant germ cells (Cromer et al., 2019; Prusicki et al., 2019; Valuchova et al., 2020). As these techniques are straightforward, they have the potential to be widely adopted in the plant meiosis field. One crucial consideration is to carefully evaluate how meiocytes can be reached and how they can be kept alive for long-term analyses spanning several hours.

### Sample Mounting and Medium Selection

Plants are sensitive to environmental conditions, including temperature, osmolarity, and humidity (Buchanan et al., 2015). Therefore, it is crucial to apply proper environmental conditions when performing a live-cell imaging experiment and to choose an appropriate culture medium to maintain tissue viability without altering its development.

#### Isolated Meiocytes

Male meiocytes, or pollen mother cells (PMCs), develop within the anthers, are sustained by a layer of tapetum cells, and are protected by the middle layer, the endothecium, and the epidermis (Figure 1). Direct observations of isolated meiocytes require that immature flower buds to be collected and opened, and their anthers removed and excised at one end. Meiocytes are then extruded by gentle squeezing from the end distal from the cut and finally transferred onto the appropriate medium.

Early attempts to culture meiocytes were published in 1967 (Ito and Stern, 1967) studying meiotic division *in vitro*. Lily microsporocytes were cultured in a culture medium whose composition was based on White’s solution (White, 1964) from zygotene through the meiotic progression (Ito and Stern, 1967; Table 1). Several challenges emerged from this first study in culturing isolated meiocytes. First, damage inflicted during meiocyte extraction severely affected the survival of the cells *in vitro*. Second, the success rate of meiocyte cell culture depended on the starting meiotic stage; meiocytes at early meiosis were delicate and suffered damage much more easily than older cells (Ito and Stern, 1967). This higher sensitivity might be related to intracellular connections among meiocytes and between meiocytes and tapetal cells, which appear to be very tight at early stages (Heslop-Harrison, 1966), resulting in rupture during dissection.

**TABLE 1:** Composition of media used for live cell imaging of isolated meiocytes, dissected anthers and flower buds + depicts addition of, * modified concentration compared to the original medium cited, – depletion of.

	Specimen	Plant species	Medium		Comments/Description	Microscope	Publication
1	Isolated meiocytes	*Lilium*	Modified White’s solution		Temperature 20 ± 1°C	Transmitted light microscope	Ito and Stern, 1967
			Component	g/L			
			Ca(NO_3_)_2_*4H_2_O	0.3			
			K NO_3_	0.08			
			KCl	0.065			
			MgSO_4_*7H_2_O	0.75			
			Na_2_SO_4_	0.2			
			NaH_2_PO_4_*H_2_O	0.019			
			MnSO_4_*4H_2_O	5 × 10^–3^			
			ZnSO_4_*7H_2_O	3 × 10^–3^			
			H_3_BO_3_	15 × 10^–4^			
			KI	75 × 10^–5^			
			CuSO_4_	1 × 10^–5^			
			Na_2_MoO_4_	1 × 10^–6^			
			Fe_2_(SO_4_)_3_	0.001			
			Glycine	0.003			
			Nicotinic acid	5 × 10^–4^			
			Thyamine	1 × 10^–4^			
			Pyridoxine	1 × 10^–4^			
			Sucrose	0.3 M	pH 5.6–5.8		

2	Isolated meiocytes	*Secale cereale* cultivar JNK	Based on 1		Temperature 21 ± 1°C	Transmitted light microscope	De La Peña, 1986
			+ MoO 3	1 × 10^–5^			
			+ Mesoinositol	0.1			
			+ Nicotinic acid	5 × 10^–4^			
			* MnSO_4_*4H_2_O	3.9 × 10^–3^			
			* KI	7.5 × 10^–5^			
			+ AlCl_3_	1 × 10^–4^			
			+ NiCl_2_*6H_2_O	1 × 10^–4^			
			* Glycine	0.051			
			+ Valine	0.05			
			+ Glutamine	0.05			
			+ Lysine	0.05			
			+ Methionine	0.05			
			+ Threonine	0.05			
			+ L-isoleucine	0.05	pH 5.8–5.9		

3	Isolated meiocytes	*Zea mays*, line W23	Based on 2		Traces elements such as Mo, Ni, and Al were not required for culturing maize meiocytes	Polarized microscope, differential interference contrast (DIC) microscope and epifluorescence microscope	Chan and Zacheus Cande, 2000
			*Sucrose	0.28–0.34 M			
			– MoO3, AlCl_3_, NiCl_2_*6H_2_O		Temperature not lower than 25°C		

4	Isolated meiocytes	*Zea mays*, line W23	Based on 2		Temperature 25 ± 1°C	Epifluorescence microscope	Yu et al., 1997
			* Sucrose	0.1 M			Nannas et al., 2016
			+ Maltose	0.1M			
			+ Guillard’s antibiotic concentrated solution	1%			
			+ *n*-propyl gallate	0.25 mM			

5	Anthers	*Agapanthus umbelatus*	Artificial Pond Water (APW)			Two-photon excitation microscope	Feijó and Cox, 2001
		*Zea mays*	NaCl	0.1 M		Two-photon excitation microscope	Sheehan and Pawlowski, 2009
			CaCl_2_	0.1 M			
			KCl	0.1 M			

6	Flower buds	*Arabidopsis thaliana*	Apex growth medium			Confocal laser scanning microscope	Prunet et al., 2016
			Murashige and Skoog basal salt mixture without vitamins	0.5 x			
			Sucrose	1%			
			Agarose	0.80%			
			Myo-inositol	0.01%			
			Nicotinic acid	0.0001%			
			Pyridoxine hydrochloride	0.0001%			
			Thiamine hydrochloride	0.001%			
			Glycine	0.0002%			
			Cytokinins (*N6*-benzyladenine)	500 nM	pH 5.8		

7	Flower buds	*Arabidopsis thaliana*	*In vitro* culture medium (Nitsch medium)			Two-photon excitation microscope	Ingouff et al., 2017
			Trehalose	5% [w/v]			
			MES-KOH at pH 5.8	0.05% [w/v]			
			Gamborg’s vitamin solution	1x			
			Agarose	8%			
							

8	Flower buds	*Arabidopsis thaliana*	Apex Culture Medium (ACM)		Temperature 21°C	Confocal laser scanning microscope	Prusicki et al., 2019
			Murashige and Skoog basal salt	0.5x			
			Sucrose	1%			
			Agarose	0.80%			
			Myo-inositol	0.01%			
			Nicotinic acid	0.0001%			
			Pyridoxine hydrochloride	0.0001%			
			Thiamine hydrochloride	0.001%			
			Glycine	0.0002%	pH 5.8		

9	Flower buds	*Arabidopsis thaliana*	Murashige and Skoog basal salt	0.5x	Temperature 21°C	Light sheet fluorescence microscope	Valuchova et al., 2020
			Sucrose	5%			
			Agarose	1%	pH 5.8		

Nonetheless, meiocytes from various species of liliaceous plants have been successfully cultured since then (Stern and Hotta, 1970; Takegami et al., 1981; Ryan, 1983). Modifications to the composition of the original medium, such as the addition of microelements, known to increase cell survival, allowed the culturing of isolated meiocytes from rye (*Secale cereale*) (De La Peña, 1986; Rueda and Vázquez, 1988; Table 1).

However, while these plants have large enough chromosomes to be viewed using transmission light microscopy, none of these species is easily genetically tractable. Hence, adapting the methods implemented for the culture of lily and rye meiocytes to a system more amenable to genetic manipulation, such as maize, was an important advance to study the molecular mechanisms underlying meiosis in plants. Living maize meiocytes were successfully cultured and remained viable from pachytene to telophase II, and progression of meiosis and chromosome segregation was monitored for 24 h by epifluorescence microscopy (Chan and Zacheus Cande, 2000).

In addition to the specific composition of the culture medium, two environmental factors are crucial for culturing maize meiocytes: (1) the osmolarity of the medium, with an emphasis on sucrose concentration [the concentration range is very narrow for maize (0.28–0.34 M) (Chan and Zacheus Cande, 2000) but varies from plant to plant (Takegami et al., 1981)]; and (2) the temperature: maize meiocytes cannot be cultured at temperatures below 25°C without causing abnormal chromosome segregation (Yu et al., 1997).

Further experiments on isolated meiocytes have only been conducted in maize, following the same culturing principles based on White’s solution (White, 1964), with a few adjustments (Table 1). For example, the sucrose concentration was lowered to 0.1 M and the medium was supplemented with 0.1 M maltose, 1% (v/v) Guillard’s antibiotic concentrated solution, and 0.25 mM *n*-propyl gallate, known to increase the longevity of maize protoplasts in culture (Yu et al., 1997). Meiocytes extruded into this medium were viable for 9 h or longer and were observed undergoing meiosis II (Yu et al., 1997). The same medium was used to support growth of maize male meiocytes while imaging live meiosis I and meiosis II by fluorescence microscopy (Higgins et al., 2016; Nannas et al., 2016; Table 1).

#### Dissected Anthers

The first microscopy study of meiosis in intact and living plant anthers was performed in African lily (*Agapanthus umbelatus*) (Feijó and Cox, 2001). Freshly isolated anthers were incubated on a minimal medium (artificial pond water [APW]) that supported tissue viability without inducing major alterations in size or morphology for up to 3 days in culture (Feijó and Cox, 2001). APW is also an optically clear isotonic solution that causes minimal light scattering; moreover, APW is a minimal medium, i.e., without sugar, and is thus less likely to become contaminated with bacteria over long time-course experiments (Sheehan and Pawlowski, 2009; Table 1).

A similar approach was successfully implemented for imaging maize meiocytes; culturing them in a microscope slide chamber containing APW to examine chromosome dynamics during meiosis prophase I ensured a viability of over 30 h (Sheehan and Pawlowski, 2009). This approach allowed the application of chemicals, agents, or drugs such as cytoskeleton-disrupting drugs (latrunculin and colchicine) and the observation of the resulting effects in living microspore mother cells (Sheehan and Pawlowski, 2009).

Imaging entire anthers offers the advantage of maintaining the developmental context of meiocytes, at least to some extent, thus limiting the influence of *in vitro* culturing on isolated meiocytes. Recently, this isolation method was applied to Arabidopsis; time-lapse movies of isolated anthers were recorded by a LCSM for over 4 h (Cromer et al., 2019). Flower buds were dissected, and undamaged anthers were transferred onto a slide topped by a spacer (0.12 mm deep) and filled with water as culturing medium. However, isolated anthers are fragile, especially at early stages when they contain meiocytes, and the dissection itself can easily stress and damage them, limiting the time span of live-cell imaging and raising the possibility that any observation reflects a stress reaction instead.

#### Flower Buds

As an alternative to isolated anthers, entire flower buds can be imaged, as recently established in Arabidopsis (Prusicki et al., 2019). Using entire flower buds is the least invasive *ex vivo* technique and thus further reduces the potential influence of sample handling before and during imaging. In the case of Arabidopsis, movies of living meiocytes of up to 30 h have been obtained (Prusicki et al., 2019).

This method is derived from the procedure previously applied for the observation of the development of emerging floral buds (Prunet et al., 2016; Table 1). Inflorescences are harvested, and all but one young flower primordium, which contains cells undergoing meiosis, are removed. The upper sepal is then removed to reveal two of the six anthers. Finally, the bud along with the pedicel and a few millimeters of the stem is embedded in Arabidopsis apex culture medium (ACM) (Table 1), kept in place with a drop of agarose, submerged in water, and imaged with an upright LCSM equipped with a water immersion objective (Prusicki et al., 2019, 2020a,b). Samples remain alive for up to 2 days with this method, allowing the analysis of the entire meiosis progression (Prusicki et al., 2019). This method also allows the addition of drugs such as oryzalin to the imaging medium; in the case of oryzalin, the effects induced by microtubule depolymerization can then be monitored in living meiocytes (Sofroni et al., 2020). Moreover, this imaging setup was also used to follow meiotic progression under heat stress, which was induced by using a heated incubation chamber surrounding the microscopic stage (De Jaeger-Braet et al., 2021).

Flower buds are also the starting material for an alternative approach of live-cell imaging for both the male and female germ cell lineage by light sheet fluorescence microscopy (LSFM) (Valuchova et al., 2020). The flower buds at a stage of interest are detached from inflorescences, and their sepals and petals are removed. To observe female meiosis, the anthers are excised, the stigma is cut off, and the valves are opened to expose the ovules attached to the septum. The dissected specimen is then embedded into capillaries containing medium with 1% (w/v) low-melting-point agarose (Valuchova et al., 2020; Table 1). Exploiting the fast image acquisition speed and the low phototoxicity and photobleaching of LSFM, long-term imaging sessions of up to 5 days are possible; three-dimensional images can also be acquired over time, allowing the introduction of a fourth dimension in the data.

While imaging entire flower buds is the method with the greatest potential, this approach is also limited in terms of specimen size. Imaging larger and/or more complex flower primordia than those of Arabidopsis, like maize, will require the development of other setups. Possible alternatives include a technique pioneered for live-cell imaging of methylation changes during Arabidopsis sporogenesis and gametogenesis. Here, the inflorescences are embedded in a solid *in vitro* culture medium (Nitsch medium), dissected with a vibratome, and observed by two-photon microscopy (Ingouff et al., 2017; Table 1).

### Reporter Lines

A prerequisite for the study of cellular dynamics during meiosis is the labeling of cellular components (organelles, chromatin, microtubules, and others) so they can be visualized by chemical staining or by fluorescently labeled fusion proteins.

Chemical staining with the nucleic acid stains SYTO 12 and DAPI (4′,6-diamidino-2-phenylindole) has been used to visualize isolated meiocytes or anthers in maize (Sheehan and Pawlowski, 2009; Higgins et al., 2016; Nannas et al., 2016). Chemical staining can be achieved on non-transgenic materials, which can be an advantage for plant species that cannot be transformed easily. However, the chemicals need to cross multiple cell layers before reaching and entering the meiocytes, making them more suited for imaging isolated cells. Protein chimeras, or fluorescent reporters, offer another route. They consist of a fusion between a fluorescent protein (FP) such as enhanced green fluorescent protein (EGFP), mVenus, or mCherry and the protein of interest. The transgenes encoding these fusions may be driven by the promoter of the gene of interest to minimize the potential for overexpression artifacts (Bastiaens and Pepperkok, 2000; DeBlasio et al., 2010). However, proteins with lower abundance might not be easily detectable during live-cell imaging; in these cases, highly active promoters like *UBIQUITIN10* (*UBQ10*) or *RIBOSOMAL PROTEIN S5A* (*RPS5A*) (Weijers et al., 2001) are sometimes preferred choices. Alternatively, tissue-specific promoters, like that of *DMC1*, are employed when the promoter of interest is too weak or not well defined (Zhao et al., 2017). Typically, the FPs are added to the N or C terminus of the protein of interest; however, inserting the fluorescent tags along the protein might be necessary if a fusion to either terminus interferes with protein function, as it does in ZIPPER1 (ZYP1) (Yang et al., 2019).

Choosing the right FP is the first step in designing any live-cell imaging assay. New FPs are constantly developed, and it is well advised to browse the literature for the latest advances in the field or check databases such as *I love GFP*^1^. Ideal FPs for live-cell imaging are as bright and as photostable as possible. However, photostability will hinder fluorescence recovery after photobleaching (FRAP) experiments. Multimeric FPs, such as the tetrameric DsRed, are brighter but are likely to produce artifacts and/or interfere with protein function through forced dimerization or multimerization of the target protein. FPs should also be selected to allow the concomitant visualization of two or even three proteins at the same time. Often a GFP or yellow fluorescent protein (YFP) variant is combined with an RFP variant due to the adequate spectral separation of their excitation and emission spectra. When selecting the right FP, another consideration is that some proteins are excited with shorter wavelengths; for example, blue light is more toxic to the cell than longer wavelengths. However, the shorter the wavelength, the higher will be the spatial resolution due to the diffraction limit of microscopy. Another challenge in plant applications is the notorious autofluorescence of plant cells from chlorophyll, lignin, and flavonoids that may interfere with the detection of the fluorescent reporter of interest.

Photo-switchable and photo-activatable FPs have recently been applied to live-cell imaging in plants, expanding the palette of available reporters. One example is the monomeric fluorescent reporter EosFP, which is irreversibly converted from a green-emitting to a red-emitting protein upon exposure to ultraviolet light. When fused to proteins with multiple subcellular localizations, EosFP allows for a color-based differentiation between individual cells and organelle subpopulations and can be adapted to tracking subcellular structures and their interactions (Mathur et al., 2012). For instance, the *35Spro:H2B-mEosFP* reporter, expressing a fusion construct between *histone H2B* and *EosFP* driven by the cauliflower mosaic virus (CaMV) 35S promoter, was transformed into tobacco (*Nicotiana tabacum*) and Arabidopsis plants to study endoreplication and changes in DNA content in living cells (Wozny et al., 2012). A second example is the photo-convertible monomeric protein Kikume Green-Red (mKikGR) variant, which was fused to HISTONE THREE RELATED10 (HTR10, with the reporter construct *HTR10pro:HTR10-mKikGR*) to study the dynamics of two identical sperm cells during fertilization in Arabidopsis (Hamamura et al., 2011).

Fluorescent reporters are not only useful as fusion proteins that inform on protein localization; they can also be designed to visualize and quantify the transcriptional response to a chemical stimulus, such as with the auxin reporter *DR5:N7-mVenus* (Table 2), used to indirectly visualize the distribution of the phytohormone during flower development (Valuchova et al., 2020). In this case, the fluorescent tag is not fused to another protein, but instead provides a visual read-out of the transcriptional activation of the synthetic *DR5* promoter by auxin (Valuchova et al., 2020).

**TABLE 2:** Fluorescent reporters expressed, or potentially expressed, in meiosis.

Cell compartment	Plant species	Construct	Meiotic specific	Meiotic phase	Publication
**Chromosome and chromatin markers**					
Histones	*Arabidopsis thaliana*	*PRO_*HTA*10_:HTA10:RFP*	No	All meiosis	Valuchova et al., 2020
		*PRO_*H*2*B*_:H2B:mRuby2*	No	All meiosis	Valuchova et al., 2020
		*PRO_35*S*_:H2B:mEosFP*	No	Not observed during meiosis	Wozny et al., 2012
		*PRO_*HTR*10_:HTR10:mKikGR*	No	Not observed during meiosis	Hamamura et al., 2011
Centromeric histones	*Zea mays*	*PRO_35S_:CenH3:YFP*	No	All meiosis	Jin et al., 2008
Telomeres	*Nicotiana Benthamiana*	*PRO_*PcUbi*4_:Sp/Sa- dCas9:eGFP/mRuby2*	No	Not observed during meiosis	Dreissig et al., 2017
		*PRO_*UBQ*6_:sgRNA-telomere*	No	Not observed during meiosis	Dreissig et al., 2017
		*PRO_*Ubi*_:dCas9:2xMS2:GFP*	No	Not observed during meiosis	Khosravi et al., 2020
		*PRO_35S_:dCas9:2xMS2:GFP*	No	Not observed during meiosis	Khosravi et al., 2020
		*PRO_*RPS5A*_:dCas9:2xMS2:GFP*	No	Not observed during meiosis	Khosravi et al., 2020
		*PRO_*Ubi*_:dCas9:3xPP7:GFP*	No	Not observed during meiosis	Khosravi et al., 2020
		*PRO_35S_:dCas9:3xPP7:GFP*	No	Not observed during meiosis	Khosravi et al., 2020
		*PRO_*RPS5A*_:dCas9:3xPP7:GFP*	No	Not observed during meiosis	Khosravi et al., 2020
DNA Methylation: CG type	*Arabidopsis thaliana*	*PRO_*HTR*5_:MBD:Venus*	No	All meiosis	Ingouff et al., 2017
DNA Methylation: CHH type	*Arabidopsis thaliana*	*PRO_*HTR*5_:SUVH9:Venus*	No	All meiosis	Ingouff et al., 2017
DNA replication	*Arabidopsis thaliana*	*PRO_*PCNA*1_:PCNA1:GFP*	No	Not observed during meiosis	Yokoyama et al., 2016
		*PRO_*PCNA*1_:PCNA1:TagRFP*	No	All meiosis, specific dots and speckles in S-phase	Valuchova et al., 2020
Cohesion	*Arabidopsis thaliana*	*PRO_*REC*8_:REC8:GFP*	Yes	Prophase, metaphase I	Prusicki et al., 2019
		*PRO_*SWI*1_:SWI1:GFP/RFP*	Yes	Leptotene	Yang et al., 2019
		*PRO_*WAPL*1_:WAPL1:GFP*	No	All meiosis	Yang et al., 2019
Chromosome axis and synaptonemal complex	*Arabidopsis thaliana*	*PRO_*ASY*1_:ASY1:GFP/RFP*	Yes	Prophase	Yang et al., 2020
		*PRO_*ASY*1_:ASY1:eYFP*	Yes	Prophase	Valuchova et al., 2020
		*PRO_*ASY*3_:ASY3:RFP*	Yes	Prophase	Yang et al., 2020
		*PRO_*ASY*4_:ASY4:eYFP*	Yes	Prophase	Chambon et al., 2018
		*PRO_*ZYP*1*b*_:ZYP1b:GFP*	Yes	Zygotene, pachytene	Yang et al., 2020
		*PRO_*PCH*2:_PCH2:GFP*	No	Prophase	Yang et al., 2020
		*PRO_*COMET*_:COMET:GFP*	No	Prophase	Balboni et al., 2020
**Cytoskeletal markers**					
Microtubules	*Zea mays*	*CFP:TUB1*	No	All meiosis	Higgins et al., 2016; Nannas et al., 2016
	*Arabidopsis thaliana*	*PRO_*UBQ14*_:GFP:TUA6*	No	All meiosis	Brownfield et al., 2015
		*PRO_*RPS*5*A*_:TagRFP:TUB4*	No	All meiosis	Prusicki et al., 2019
		*PRO_*RPS*5*A*_:TagRFP:TUA5*	No	All meiosis	Prusicki et al., 2019
Phragmoplast	*Arabidopsis thaliana*	*PRO_*MAP*65–3_:GFP:MAP65-3*	No	Cytokinesis	Sofroni et al., 2020
Actin	*Arabidopsis thaliana*	*PRO_35*S*_:Lifeact:Venus*	No	Not observed during meiosis	Era et al., 2009
Actin related protein	*Arabidopsis thaliana*	*PRO_*ARP*6_:ARP6:YFP*	No	Prophase, female meiosis	Qin et al., 2014
**Organelle reporters**					
Golgi	*Arabidopsis thaliana*	*PRO_*JAS*_: JAS:GFP*	No	All meiosis, organeller band	Brownfield et al., 2015
		*PRO_*UBQ*14_:JAS:GFP*	No	All meiosis, organeller band	Brownfield et al., 2015
Nucelar envelope	*Arabidopsis thaliana*	*PRO_*SUN*1_:SUN1:GFP*	No	All meiosis	Varas et al., 2015
		*PRO_*SUN*2_:SUN2:GFP*.	No	All meiosis	Varas et al., 2015
Plasma membrane	*Arabidopsis thaliana*	*PRO_*SYP*132_:GFP:SYP132*	No	All meiosis	Sofroni et al., 2020
**Cell cycle reporters**					
CDKs	*Arabidopsis thaliana*	*PRO_*CDKA;*1_:CDKA;1:mVenus*	No	All meiosis	Sofroni et al., 2020
		*PRO_*CDKA;*1_:CDKA;1:mTurquoise*	No	All meiosis	Sofroni et al., 2020
		*PRO_*CDKD;1*_:CDKD;1:mVenus*	No	All meiosis	Sofroni et al., 2020
		*PRO_*CDKD;2*_:CDKD;2:mVenus*	No	All meiosis	Sofroni et al., 2020
		*PRO_*CDKD;3*_:CDKD;3:mVenus*	No	All meiosis	Sofroni et al., 2020
Cyclin	*Arabidopsis thaliana*	*PRO_*CYB*3;1_:CYCB3;1:GFP*	No	Prophase, metaphase I	Sofroni et al., 2020
Checkpoints	*Arabidopsis thaliana*	*PRO_*BORR*_:BORR:GFP*	No	Anaphase I, II and cytokinesis	Komaki et al., 2020
		*PRO_*INCENP*_:GFP:INCENP*	No	Anaphase I, II and cytokinesis	Komaki et al., 2020
**Homologous recombination reporters**					
Double strand breaks	*Arabidopsis thaliana*	*PRO_*RAD51:*_RAD51:GFP*	No	Not observed during meiosis	Da Ines et al., 2013
	*Arabidopsis thaliana*	*PRO_*RBR:*_mCherry:RBR*	No	Not observed during meiosis	Biedermann et al., 2017

#### Chromosome and Chromatin Markers

**FIGURE 2:**
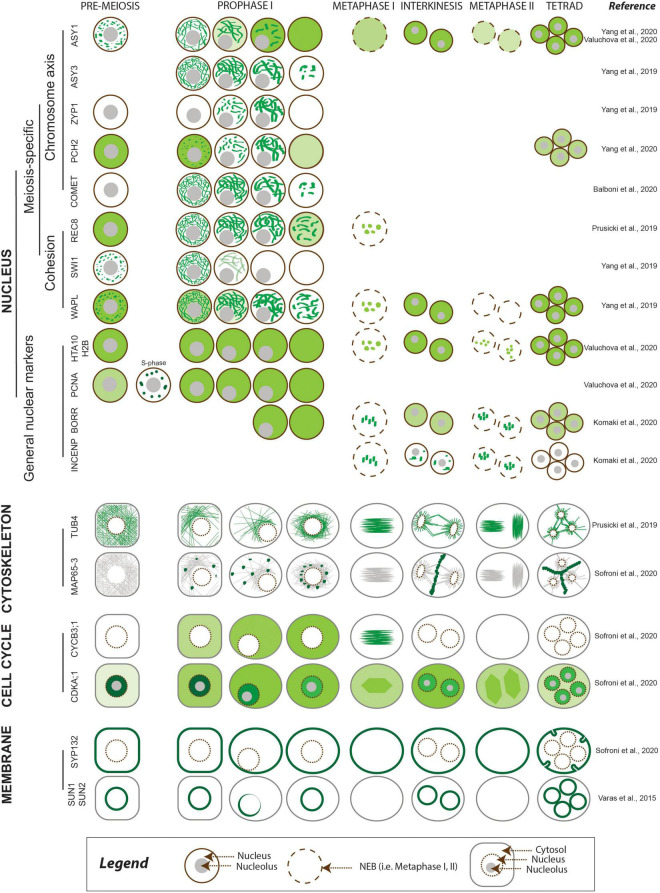
Meiotic localization patterns of meiosis-specific and non-meiosis-specific plant proteins reported to date. The abundance of the protein of interest is depicted using different shades of green, corresponding to the relative intensity of the signal. The nucleus is outlined in brown, with other cellular compartments shown in gray.

Meiosis-specific events including pairing, recombination, assembly, and disassembly of the SC take place in the nucleus. Dissecting their dynamics requires nuclear markers (reviewed in Wozny et al., 2012) and histone markers such as the histone H2A HTA10 and the histone H2B HTB9 (Valuchova et al., 2020) to highlight chromatin, as they are present throughout meiosis (Figure 2 and Table 2). The development of reporters encoding meiosis-specific proteins may also be needed. To date, several functional meiosis-specific reporter lines have been generated and used in live-cell imaging experiments or *in vivo* localization studies: markers for the cohesin complex (REC8 [RECOMBINATION8], SWI1 [SWITCHING DEFICIENT1], and WAPL [WINGS APART-LIKE]) (Figure 2 and Table 2; Prusicki et al., 2019; Yang et al., 2019) and markers for the chromosome axis, synaptonemal complex components, and their regulators ASYNAPTIC1 (ASY1), ASY3, ASY4, ZYP1b, PACHYTENE CHECKPOINT2 (PCH2), and COMET (Figure 2 and Table 2; Chambon et al., 2018; Yang et al., 2019, 2020; Balboni et al., 2020; Valuchova et al., 2020). These reporters have been instrumental in unraveling the dynamics of chromosomes in meiotic cells and in elucidating more complex cellular mechanisms, such as the prophase pathway of cohesin removal in plants and the regulation of the meiotic chromosome axis (Cromer et al., 2019; Yang et al., 2019, 2020; Balboni et al., 2020).

Centromeres are typically fluorescently tagged using CENTROMERIC HISTONE H3 (CENH3) and constitute a visual marker to determine gametophytic and somatic ploidy in Arabidopsis (De Storme et al., 2016; Liu et al., 2017). A maize YFP-tagged centromere line derived from the *ZmCENH3* cDNA sequence (35S*pro:CenH3:YFP*) has been reported, but it has yet to be used in meiotic studies (Jin et al., 2008; Table 2). With respect to telomeres, live-cell CRISPR-Cas9-based imaging using EGFP or mRuby2 fused to the CRISPR-associated nuclease Cas9 allowed the visualization of telomeric regions *in vivo* in *Nicotiana benthamiana* leaves by harnessing the intrinsic ability of the nuclease to recognize palindromic repeats (Dreissig et al., 2017; Khosravi et al., 2020; Table 2). However, to date telomeres have not been successfully observed by live-cell imaging in any plant during meiosis.

Finally, levels and changes in DNA methylation during meiosis and gametophyte development in Arabidopsis are accessible via live-cell imaging of specific reporters for CG or non-CG methylation marks, such as *HTR5pro:MBD-Venus* (a fusion between a methyl-CpG-binding domain [MBD] and Venus under the control of the histone H3 *HTR5* promoter) and *HTR5pro:SUVH9-Venus* (a fusion between SU(VAR)3-9 HOMOLOG 9 and Venus), respectively (Table 2; Ingouff et al., 2017).

#### Cytoskeletal Markers

The microtubule cytoskeleton plays an essential role during cell divisions and undergoes dramatic changes over the course of meiosis. Therefore, fluorescent reporters for cytoskeletal elements provide important information on meiotic progression. A maize reporter line consisting of a fusion protein between the cyan fluorescent protein (CFP) and TUBULIN BETA, *ECFP-TUB* (Table 2), was used to study spindle assembly (Higgins et al., 2016) and the positioning of the division plane during metaphase I and II (Nannas et al., 2016). Similar reporters were developed in Arabidopsis with TUBULIN alpha (TUA5) and TUBULIN beta (TUB4) fused to TagRFP (*RPS5Apro:TagRFP-TUB4* and *RPS5Apro:TagRFP-TUA5*) and driven by the *RPS5A* promoter (Figure 2 and Table 2). These reporters made it possible to describe the spatiotemporal dynamics of microtubule configurations during meiosis (Prusicki et al., 2019) and revealed microtubule dynamics anomalies in mutants with lower CDK activity such as *tam* (*tardy asynchronous meiosis*) or the *cdka;1 cdkd;3* double mutant (Prusicki et al., 2019; Sofroni et al., 2020).

A GFP-tagged tubulin reporter driven by the *UBIQUITIN14* promoter (*UBQ14pro:GFP-TUA6*) (Table 2) allowed the visualization of tubulin in fixed Arabidopsis anthers (Brownfield et al., 2015). Furthermore, motor proteins such as microtubule-associated proteins (MAPs) are emerging as powerful reporters to characterize meiotic cytokinesis, as is the case of the reporter *MAP65-3pro:GFP-MAP65-3* (Figure 2 and Table 2; Sofroni et al., 2020).

In contrast to microtubules, the regulation of actin filaments has not been extensively studied in plants, even though their relevance during cell division is well known (Rasmussen et al., 2013). FP-tagged Lifeact (Era et al., 2009), a yeast-derived actin-binding peptide, has provided a means to visualize actin filaments during pollen tube growth (Vogler and Sprunck, 2015; Liao and Weijers, 2018) and cell division (Wu and Bezanilla, 2014; Kimata et al., 2016). Of the naturally accumulating actin-binding proteins, only ACTIN RELATED PROTEIN6 (ARP6) fused to YFP was observed during meiosis, with a focus on ovules undergoing megasporogenesis (Qin et al., 2014; Table 2).

#### Cell Cycle Reporters

Markers able to differentiate phases of the cell cycle are very powerful tools when studying cell division and cell cycle progression (Echevarría et al., 2021). For example, reporters based on *PROLIFERATING CELL NUCLEAR ANTIGEN1* (*PCNA1*) (*PCNA1pro:PCNA1-sGFP* and *PCNA1pro:PCNA1-TagRFP*) allow distinction between pre-meiotic and early meiotic stages, as PCNA;1 accumulates in small speckles during early S-phase and in large foci in late S-phase, while it shows a diffuse localization throughout the nucleoplasm after S-phase (Figure 2 and Table 2; Yokoyama et al., 2016; Valuchova et al., 2020).

CDKs (in particular CDKA;1) and cyclins are among the main regulators of meiosis (Harashima et al., 2013, 2016; Wijnker and Schnittger, 2013), and several fluorescent reporters have already been developed to explore their dynamics. For example, the reporters *CDKA1;1pro:CDKA;1-mVenus* and *CDKA1;1pro:CDKA;1-mTurquoise* revealed the accumulation pattern of CDKA;1, which shows high abundance in the nucleus at early prophase, followed by a decrease at mid and late prophase, as reflected by the observed increase in fluorescence from the cytosol (Figure 2 and Table 2; Sofroni et al., 2020; Yang et al., 2020). Furthermore, the dynamics of CDKA;1 accumulation have been analyzed concomitantly with those of its regulators, such as CDK-activating kinases, which in Arabidopsis consist of cyclins like CYCB3;1 and D-type CDKs. CYCB3;1 exhibited a distinct association with the first meiotic spindle at metaphase I, making it a good reporter for the spindle (Sofroni et al., 2020; Figure 2 and Table 2).

Other cell cycle reporters are based on kinetochore components: the chromosome passenger complex (CPC) proteins *BORRpro:BORR-GFP* (encoding a fusion between the plant Borealin-like protein BOREALIN-RELATED and GFP) and *INCENPpro:GFP-INCENP* (for INNER CENTROMERE PROTEIN) are characterized by a highly dynamic localization pattern from nuclear envelope breakdown until the onset of anaphase (Komaki et al., 2020; Figure 2 and Table 2). Additional reporters for kinetochore components such as spindle assembly checkpoint (SAC) proteins are available and awaiting analysis during meiosis (Komaki and Schnittger, 2017). These reporters can also be used to study chromosome dynamics (see above).

#### Membrane and Organellar Markers

During meiotic progression, membranes and other compartments, e.g., the nuclear envelope, organelles, and phragmoplast components, play pivotal roles in chromosome pairing, spindle positioning, and successful cytokinesis (Murphy et al., 2014; Brownfield et al., 2015; Varas et al., 2015). Tracking membrane and organelle behavior *in vivo* thus has the potential to reveal interesting aspects of their function and regulation. To date, published work in this area of research is limited (Brownfield et al., 2015; Varas et al., 2015). Varas et al. (2015) visualized the nuclear envelope in Arabidopsis anthers using the fluorescent reporters *SUN1pro:SUN1-GFP* and *SUN2pro:SUN2-GFP* (Figure 2 and Table 2). SUN1 and SUN2 (for Sad1/UNC-84) are two components of the protein complex that form a bridge over the nuclear membrane and link elements from the nucleoplasm to the cytoskeleton (Varas et al., 2015). Brownfield et al. (2015) used the reporters *JASpro:JAS-GFP* and *UBQ14pro:JAS-GFP* (encoding a fusion protein between the unknown protein JASON [JAS] and GFP) (Table 2) to study the behavior of the organellar band, which forms after the first meiotic division. JAS was reported to localize to endomembrane vesicles involved in Golgi trafficking (Brownfield et al., 2015). Various FP-based reporters for the Golgi apparatus, the endoplasmic reticulum, peroxisomes, mitochondria, the plasma membrane, and the tonoplast have been introduced in Arabidopsis, tobacco, and rice but have yet to be analyzed during meiosis (Brownfield et al., 2015; Dangol et al., 2017; Ito et al., 2018). Finally, the *SYP132pro:GFP-SYP132* reporter line (encoding GFP fused to SYNTAXIN OF PLANTS132 [SYP132]) was used to fluorescently label the plasma membrane in male meiocytes and revealed an outside–in direction of membrane invagination during male meiosis, whether cytokinesis was simultaneous or successive (Figure 2 and Table 2; Sofroni et al., 2020).

#### Recombination Markers

So far, very few reporters have been generated in plants to monitor recombination. The above-mentioned chromosome axis markers and reporters for the SC allow a broad temporal assignment of recombination processes based on previous annotation of each stage with immunolocalization techniques, for instance, labeling double-strand break (DSB) formation and localizing ASY1 to the axis roughly within a similar time window (Sanchez-Moran et al., 2007). Likewise, the reduction of HOMOLOG OF HUMAN HEI 10 (HEI10) foci from about 100 to around 10 in male meiosis is indicative of crossover (CO) formation and corresponds to the duration of the complete decoration of the chromosome axis by ASY1 to its partial removal from chromosome arms (Chelysheva et al., 2012). Moreover, the presence of the SC coincides with CO maturation, as visualized by the incorporation of the transverse filament component ZYP1.

However, a direct visualization of components of the recombination machinery has seldom been achieved. There is a lag in generating recombination reporters for proteins directly involved in DSB and CO formation and resolution such as SPORULATION11 (SPO11), DMCI1, HEI10, MutL HOMOLOG1 (MLH1), MutS HOMOLOG4 (MSH4), and MMS and UV SENSITIVE81 (MUS81); these proteins are instead extensively used in immunolocalization experiments in fixed samples. Although functional reporters for the Arabidopsis homologous recombination repair components RETINOBLASTOMA RELATED1 (RBR1) and RAD51 (*RBR1pro:mCherry-RBR1* and *RAD51pro:RAD51-GFP*) are available (Chen et al., 2011; Da Ines et al., 2013; Biedermann et al., 2017), they have not yet been characterized or assessed for progression of meiotic recombination.

## Aspects of Plant Meiosis Studied by Live-Cell Imaging

### Time Courses

A change in meiotic duration, or in the duration of specific meiotic stages, is one of the main phenotypic alterations of plants exposed to suboptimal environmental conditions, such as high or low temperatures (reviewed in Bennett, 1971, 1977; Bomblies et al., 2015). Furthermore, the chronology of meiotic stages is often altered in meiotic mutants, such as in the maize mutant *pam1* (*plural abnormalities in meiosis1*) (Golubovskaya et al., 2002) and the Arabidopsis *tam* (Magnard et al., 2001; Prusicki et al., 2019), *msh4* (Higgins et al., 2004), *mlh3* (Jackson et al., 2006), and *pans1*Δ*D* (*patronus1*) mutants (Cromer et al., 2019) as well as plants expressing a dominant negative version of RAD51 (Singh et al., 2017). Accordingly, time courses of plant meiosis have been performed since the late 1960s using methods based on DNA labeling first with radioactive compounds (Ekberg and Eriksson, 1965; Lindgren et al., 1969; works from Bennett, reviewed in Bennett, 1971, 1977) and later with the modified thymine analogs 5-bromo-2′-deoxyuridine (BrdU) (Armstrong et al., 2003; Higgins et al., 2004; Jackson et al., 2006; Sanchez-Moran et al., 2007) and 5-ethynyl-2′-deoxyuridine (EdU) (Higgins et al., 2012; Stronghill et al., 2014; Varas et al., 2015; Singh et al., 2017) (an overview of time courses for plant meiosis in wild-type backgrounds is presented in Table 3).

**TABLE 3:** Duration of meiosis in plants.

*MONOCOT*																			

**Plant species**	**Publication**	**Method**	**Temperature**	**Overall duration of male meiosis**	***S-phase/G2***	***Leptotene***	***Zygotene***	***Pachytene***	***Diplotene***	***Diakinesis***	***Metaphase I***	***Anaphase I***	***Telophase I***	***Interkinesis***	***Prophase II***	***Metaphase II***	***Anaphase II***	***Telophase II***	***Tetrads***
*Allium cepa*	Vasil, 1959	Aceto-carmine staining	NOT GIVEN	96 h	//	//	//	//	//	//	//	//	//	//	//	//	//	//	//

*Convallaria majalis*	Reported in Bennett, 1977	Not available	20°C	72 h	//	//	//	//	//	//	//	//	//	//	//	//	//	//	//

*Dasypyrum villosum*	Stefani and Colonna, 1996	Aceto-orcein staining	Field in May	35 ± 1.7 h	//	15.5 h					10.5 h							8 h	//
			Field in July	22 ± 2 h	//	12 h					6 h							3.5 h	//
			5°C	136 ± 14.4 h	//	69.5 h					46 h							22 h	//
			10°C	88 ± 5.3 h	//	48 h					20.5 h							20 h	//
			20°C	29 h	//	14 h					10 h							5 h	//
			28°C	21 ± 0.7 h	//	12.5 h					5.5 h							4.5 h	//
			35°C	17 ± 0.7 h	//	10 h					5 h							3 h	//

*Endymion nonscriptus*	Wilson, 1959	Aceto-carmine staining	0°C	864 h	//	//	//	//	//	//	//	//	//	//	//	//	//	//	//
			5°C	360 h	//	//	//	//	//	//	//	//	//	//	//	//	//	//	//
			10°C	168 h	//	//	//	//	//	//	//	//	//	//	//	//	//	//	//
			15°C	84 h	//	//	//	//	//	//	//	//	//	//	//	//	//	//	//
			20°C	48 h	//	//	//	//	//	//	//	//	//	//	//	//	//	//	//
			25°C	30 h	//	//	//	//	//	//	//	//	//	//	//	//	//	//	//
			30°C	20 h	//	//	//	//	//	//	//	//	//	//	//	//	//	//	//
			15–21°C	66 h	//	//	//	//	//	//	//	//	//	//	//	//	//	//	//

*Fritillaria meleagris*	Barber, 1942	Acetocarmine staining	12–15°C	400 h APPROXIMATE	//	//	//	//	//	//	//	//	//	//	//	//	//	//	//

*Hordeum vulgare:* unspecified variety	Lindgren et al., 1969	Aceto-orcein staining	NOT GIVEN	//	5.30%			32.60%	19.90%										23.70%
				//	//	//	//	//	19.50%	8%	24.80%	2.40%	4.60%	13.00%	1.50%	7.20%	5.40%	13.60%	
				//	//	//	//	//	17.90%	7.50%	24.10%	2.50%	4.70%	13.40%	1.60%	7.60%	5.80%	14.90%	
				//	3 days after the first anaylsed material all the anthers had microspores → all meiocytes terminated meiosis. One “spikelet unit” see Ekberg and Eriksson (1965), is less than 16 h → shorter stages less than 1 h														

*Hordeum vulgare*: Sultan	Bennett and Finch, 1971	Feulgen staining	20°C	39.4 h	//	12 h	9 h	8.8 h	2.2 h	36 min	1.6 h	30 min	30 min	dyad stage: 2 h		1.2 h	30 min	30 min	8 h

*Hordeum vulgare:* Ymer	Finch and Bennett, 1972	Thymidine pulse + autoradiography	20°C	39 h	//	11.5 h	9 h	9.3 h	1.9 h	36 min	1.6 h	30 min	30 min	dyad stage: 1.7 h		1.5 h	30 min	30 min	>7 h

*Hordeum vulgare:* Ymer 4X	Finch and Bennett, 1972	Thymidine pulse + autoradiography	20°C	31 h	//	9 h	7 h	7 h	1.8 h	30 min	1.5 h	24 min	24 min	dyad stage: 1.5 h		1 h	24 min	24 min	>6 h

*Hordeum vulgare:* Morex	Higgins et al., 2012	BrdU and EdU labeling	22°C	43 h	13 h	43 h													
			30°C	43 h	9 h	43 h													

*Lilium candidum*	Reported in Bennett, 1977	Not available	NOT GIVEN	168 h	//	//	//	//	//	//	//	//	//	//	//	//	//	//	//

*Lilium henryi*	Reported in Bennett, 1977	Not available	NOT GIVEN	170 h	//	//	//	//	//	//	//	//	//	//	//	//	//	//	//

*Lilium hybrid: Black Beuty*	Bennett et al., 1975	Fuchsin staining	20°C	264 h	//	//	//	//	//	//	//	//	//	//	//	//	//	//	//

*Lilium hybrid: Sonata*	Bennett et al., 1975	Fuchsin staining	20°C	180 h	//	//	//	//	//	//	//	//	//	//	//	//	//	//	//

*Lilium longiflorum: variety unspecified*	Marquardt, 1937	Aceto-carmine staining	NOT GIVEN	96 h	//	//	//	//	//	//	//	//	//	//	//	//	//	//	//

*Lilium longiflorum:* Nellie White	Ito and Stern, 1967	Autoradiography	22°C	ca192 h	//	//	//	//	//	//	//	//	//	//	//	//	//	//	//

*Lilium longiflorum:* Croft	Taylor and McMaster, 1953	Autoradiography	23°C	ca192 h	//	//	//	//	//	//	//	//	//	//	//	//	//	//	//

*Lilium longiflorum:* Floridii	Erickson, 1948	Aceto-orcein staining	NOT GIVEN	ca240h	//	//	//	//	//	//	//	//	//	//	//	//	//	//	//

*Ornithogalum virens*	Church and Wimber, 1969	Thymidine pulse + autoradiography	18°C	72 h - APPROXIMATE	//	//	//	//	//	//	//	//	//	//	//	//	//	//	//

*Secale cereale*	Bennett et al., 1971, 1972; Bennett and Smith, 1972	Feulgen staining	15°C	88 h	//	//	//	//	//	//	//	//	//	//	//	//	//	//	//
			20°C	51 h	//	20 h	11.4 h	8 h	1 h	36 min	2 h	1 h	1 h	dyads: 2.5 h		1.7 h	1 h	1 h	//
			25°C	39 h	//	//	//	//	//	//	//	//	//	//	//	//	//	//	//

*Secale cereale 4X*	Bennett and Smith, 1972	Feulgen staining	20°C	38 h	//	13 h	9 h	6.4 h	1 h	36 min	1.8 h	42 min	42 min	dyad stage: 2 h		1.4 h	42 min	42 min	//

*Tradescantia paludosa*	Reported in Bennett, 1977	Not available	NOT GIVEN	126 h	//	//	//	//	//	//	//	//	//	//	//	//	//	//	//

*Tradescantia reflexa*	Reported in Bennett, 1977	Not available	NOT GIVEN	144 hs	//	//	//	//	//	//	//	//	//	//	//	//	//	//	//

*Trillium erectum*	Hotta and Stern, 1963	Autoradiography	1°C	2160 h	//	//	//	//	//	//	//	//	//	//	//	//	//	//	//
	Hotta and Stern, 1963	Autoradiography	2°C	1680 h	//	//	//	//	//	//	//	//	//	//	//	//	//	//	//
	Kemp, 1964	Propronio-carmine	5°C	960 h	//	//	//	//	//	//	//	//	//	//	//	//	//	//	//
	Ito and Stern, 1967	Autoradiography	15°C	288 h	//	//	//	//	//	//	//	//	//	//	//	//	//	//	//

**MONOCOT**																			

**Plant species**	**Publication**	**Method**	**Temperature**	**Overall duration of male meiosis**	***S-phase/G2***	***Leptotene***	***Zygotene***	***Pachytene***	***Diplotene***	***Diakinesis***	***Metaphase I***	***Anaphase I***	***Telophase I***	***Interkinesis***	***Prophase II***	***Metaphase II***	***Anaphase II***	***Telophase II***	***Tetrads***

*Triticale turgidum: durum*	Bennett and Kaltsikes, 1973	Feulgen/aceto-carmine staining	20°C	31 h	//	23.7 h					7.5 h								//

*Triticale: genotype A (CS/K-TA)*	Bennett and Smith, 1972	Feulgen/aceto-carmine staining	20°C	21 h	//	7.5 h	3 h	2.25 h	1 h	30 min	1.75 h	30 min	30 min	dyad stage: 1.5 h		1.25 h	30 min	30 min	//

*Triticale: genotype B (CS/Pet-TA)*	Bennett and Smith, 1972	Feulgen/aceto-carmine staining	20°C	22 h	//	15.5 h					6.5 h								//

*Triticale: Rosner*	Bennett and Smith, 1972	Feulgen/aceto-carmine staining	20°C	34 h	//	26.5 h					7.5 h								//

*Triticum diococcum 4X*	Bennett and Smith, 1972	Feulgen/aceto-carmine staining	20°C	30 h	//	22.5 h					7.5 h								//

*Triticum aestivum x Secale cereale*	Bennett et al., 1974	Feulgen/aceto-carmine staining	20°C	35.5 h	//	28 h					7.5 h								10 h

*Triticum aestivum: Chinese Spring*	Bennett et al., 1971, 1972	Thymidine pulse + autoradiography	15°C	43 h	//	//	//	//	//	//	//	//	//	//	//	//	//	//	//
			20°C	24 h	//	10.4 h	3.4 h	2.2 h	36 min	24 min	1.6 h	30 min	30 min	dyad stage: 2 h		1.4 h	30 min	30 min	//
			25°C	18 h	//	//	//	//	//	//	//	//	//	//	//	//	//	//	//

*Triticum aestivum: Holdfast*	Reported in Bennett, 1977	Not available	15°C	45 h	//	//	//	//	//	//	//	//	//	//	//	//	//	//	//
			20°C	24 or 25 h	//	//	//	//	//	//	//	//	//	//	//	//	//	//	//

*Triticum monococcum*	Bennett and Smith, 1972	Feulgen/aceto-carmine staining	20°C	42 h	//	34 h					8 h								//

*Tulbaghia violacea*	Reported in Bennett, 1977	Not available	20°C	130 h	//	//	//	//	//	//	//	//	//	//	//	//	//	//	//

*Zea mays*	Hsu et al., 1988	Aceto-carmine staining	NOT GIVEN	119.1 h	//	43 h	31 h	12.2 h	7.1 h	7.2 h	4.4 h	1.6 h	1.6 h	1.8 h	0.4 h	3.9 h	2.1 h	2.8 h	//
	Yu et al., 1997	Live cell imaging	25 ±1°C	Meiosis II: 5 h	//	//	//	//	//	//	//	//	//	2.5 h	1.5 h		1 h		//
	Nannas et al., 2016	Live cell imaging	NOT GIVEN	Anaphases: 12 min	//	//	//	//	//	//	//	12.7 ± 3.2 min	//	//	//	//	11 ± 3.7 min	//	//

**DICOTS**																			

**Plant species**	**Publication**	**Method**	**Temperature**	**Meiosis duration overall**	***S-phase/G2***	***Leptotene***	***Zygotene***	***Pachytene***	***Diplotene***	***Diakinesis***	***Metaphase I***	***Anaphase I***	***Telophase I***	***Interkinesis***	***Prophase II***	***Metaphase II***	***Anaphase II***	***Telophase II***	***Tetrads***

*Alliaria petiolata*	Reported in Bennett, 1977	Not available	NOT GIVEN	24 h	//	//	//	//	//	//	//	//	//	//	//	//	//	//	//

*Anthirrium majus*	Reported in Bennett, 1977	Not available	NOT GIVEN	24 to 34 h	//	//	//	//	//	//	//	//	//	//	//	//	//	//	//

*Arabidopsis thaliana:* Ws WT	Armstrong et al., 2003	BrdU labeling	18.5°–20°C	33 h	9 h	6 h	15.3 h		2.7 h										//

*Arabidopsis thaliana:* Ler WT	Stronghill et al., 2014	EdU labeling	21°C	29 h	7 h	5 h	6 h	10 h	1 h	//	//	//	//	//	//	//	//	//	//

*Arabidopsis thaliana:* Col-0 WT	Sanchez-Moran et al., 2007	BrdU labeling	NOT GIVEN	32 h	10 h	7 h	12 h		//	3 h									//
	Prusicki et al., 2019	Live cell imaging	21°C	26 h (from late leptotene)	//	>1.5 h	6 h	9.5 h	3 h		1 h		1 h		4 h				//
	Valuchova et al., 2020	Live cell imaging	21°C	47 h	90 min/3.5 h	//				1 h				//	50–90 min				//
	De Jaeger-Braet et al., 2021	Live cell imaging	21°C	21.2 h (from late leptotene to anaphase II)	//	14 h*		6 h**			47 min		52 min			46 min		3.6 h	//
			30°C/1 week	18.1 h (from late leptotene to anaphase II)	//	10.1 h*		6.3 h**			39 min		45 min			37 min		4.2 h	//
			30°C heat-shock	16.1 h (from late leptotene to anaphase II)	//	9.3 h*		6.1 h**			32 min		47 min			29 min		3.5 h	//
			34°C heat-shock	18.1 h (from late leptotene to anaphase II)	//	7.1 h*		8.7 h**			34 min		59 min			24 min		//	//

**DICOTS**																			

**Plant species**	**Publication**	**Method**	**Temperature**	**Overall duration of male meiosis**	***S-phase/G2***	***Leptotene***	***Zygotene***	***Pachytene***	***Diplotene***	***Diakinesis***	***Metaphase I***	***Anaphase I***	***Telophase I***	***Interkinesis***	***Prophase II***	***Metaphase II***	***Anaphase II***	***Telophase II***	***Tetrads***

*Beta vulgaris*	Reported in Bennett, 1977	Not available	20°C	24 h	//	//	//	//	//	//	//	//	//	//	//	//	//	//	//

*Capsella bursa-pastoris*	Reported in Bennett, 1977	Not available	NOT GIVEN	18 h	//	//	//	//	//	//	//	//	//	//	//	//	//	//	//

*Haplopappus gracilis*	Reported in Bennett, 1977	Not available	NOT GIVEN	24–36 h	//	//	//	//	//	//	//	//	//	//	//	//	//	//	//

*Lycopersicum esculentum (Solanum lycopersicum)*	Reported in Bennett, 1977	Not available	20°C	24–30 h	//	//	//	//	//	//	//	//	//	//	//	//	//	//	//

*Lycopersicum peruvianum*	Pacini and Cresti, 1978	Uranyl acetate staining	NOT GIVEN	Prophase 12 h	//	12 h					//	//	//	//	//	//	//	//	//

*Petunia hybrida*	Izhar and Frankel, 1973	Fixed anthers/staining not specified	15–17°C night/25–30°C day	16 h	4 h		2 h	2 h	1 h		2 h		1 h	1 h				3 h	12 h

*Pisum sativum*	Reported in Bennett, 1977	Not available	20°C	30 h	//	//	//	//	//	//	//	//	//	//	//	//	//	//	//

*Veronica chamaedrys*	Reported in Bennett, 1977	Not available	NOT GIVEN	20 h	//	//	//	//	//	//	//	//	//	//	//	//	//	//	//

*Vicia faba*	Reported in Bennett, 1977	Not available	NOT GIVEN	72 to 96 h	//	//	//	//	//	//	//	//	//	//	//	//	//	//	//

*Vicia sativa*	Reported in Bennett, 1977	Not available	20°C	24 h	//	//	//	//	//	//	//	//	//	//	//	//	//	//	//

**GYMNOSPERM**																			

Pinus laricio	Chamberlain, 1935 (Reported in Izhar and Frankel, 1973)	Not available	NOT GIVEN	3 months	//	//	//	//	//	//	//	//	//	//	//	//	//	//	//

*//depicts not calculated or not specified data,*

* inlcudes early pachytene,

** includes only late pachytene - diplotene.

In time-course experiments, the length of meiotic phases is determined by measuring the time between the labeling pulse (meiotic S-phase) and the appearance of marked chromosomes at specific stages. For instance, labeled chromosomes with a zygotene conformation are detected in Arabidopsis starting from 18 h after a pulse, while it takes 30 h after a pulse to label chromosomes from diakinesis to telophase II, indicating that the zygotene–pachytene interval lasts for about 12 h in Arabidopsis (Sanchez-Moran et al., 2007).

However, this method is laborious and relies on the efficiency of chromosome spreads in combination with immunocytochemistry. Thus, sample sizes are typically small and preclude a quantitative analysis of the resulting data. For the same reason, it is also difficult to obtain reliable estimates for heterogeneous cell populations, as for example in the *tam* mutant background. Likewise, short phases, such a meiosis II, which lasts approximately 3 h in Arabidopsis, are difficult to analyze with this method (Armstrong et al., 2003; Sanchez-Moran et al., 2007; Stronghill et al., 2014).

By contrast, live-cell imaging techniques allow the direct observation of individual cells while they undergo meiosis. Moreover, at least in the case of male meiocytes, several samples can be analyzed in one scan, making it possible, though still tedious, to obtain statistically robust sample sizes by scanning several anthers. The first dataset to temporally resolve meiotic phases by live-cell imaging was collected on maize meiocytes observed from metaphase I to telophase II (prometaphase I–metaphase I, up to 60 min; anaphase I, up to 30 min; interkinesis, up to 150 min; prometaphase II–metaphase II, up to 90 min; anaphase II/telophase II, up to 60 min) (Table 3; Yu et al., 1997, 1999). Likewise, it was possible to determine the duration of these phases in Arabidopsis from zygotene onward (zygotene, 6 h; pachytene, 9.5 h; diplotene and diakinesis, 3 h; metaphase I and anaphase I, 1 h; telophase and interkinesis, 1 h; second meiotic division, 4 h) (Table 3; Prusicki et al., 2019).

In Arabidopsis, meiotic time courses measured by live-cell imaging recapitulate the results obtained by pulse labeling experiments for an overall duration of meiosis of about 26 h (Table 3; Prusicki et al., 2019). Live-cell imaging revealed then that an increase of the ambient temperature to 30°C resulted in an acceleration of most but not all meiotic phases in Arabidopsis (De Jaeger-Braet et al., 2021). For instance, the duration of metaphase I to anaphase I was shortened from nearly 1 h to approximately 0.5 h at 30°C. An additional temperature raise to 34°C sped up specific phases of meiosis even further, e.g., late leptotene to early pachytene lasted approximately 14 h at 21°C, 9 h at 30°C and only 7 h at 34°C. At 34°C, however, pachytene to diakinesis was considerably delayed from 6 h at 21°C and 30°C to almost 9 h at 34°C. This delay, together with genetic and cell biological data, indicated the presence of a recombination checkpoint in plants (see below) (De Jaeger-Braet et al., 2021).

Obviously, the choice of reporters will limit the amount of information retrieved. In the case of the time course published by Prusicki et al. (2019), the use of REC8 and TUBULIN as cellular markers offered sufficient cellular criteria (morphological resolution) to dissect meiosis from prophase I until telophase II, although it was not possible to calculate the timing of S-phase or G2 phase, or the exact moment of meiosis onset. To obtain information on these early phases, Valuchova et al. (2020) used the *PCNA-TagRFP* reporter, which allowed the determination of the lengths of late S-phase and G2 phase to be 90 min and 3.5 h, respectively, much shorter durations than previously estimated (Table 3).

Live-cell imaging can also help analyze mutants or situations in which not all meiocytes behave similarly: to distinguish between populations of meiocytes whose progression is arrested, or with a population of meiocytes that progress at various rates through meiosis and will eventually exit meiosis after a prolonged time, as exemplified by *tam* and the weak loss-of-function allele *smg7-1* of *SUPPRESSOR WITH MORPHOGENETIC EFFECTS ON GENITALIA7*, which encodes a factor involved in RNA decay. In the case of *tam* mutants, defective in a CDKA;1 cyclin cofactor, meiocytes show prolonged late-pachytene/diplotene stages, lasting 3–5 h longer than in the wild type. Notably, different populations of meiocytes were identified with distinct microtubules structures (Prusicki et al., 2019). Live-cell imaging of *smg7-1* revealed that the previously described arrest at anaphase II occasionally results from a regression of cells that already entered telophase II. Such a regression was not observed in fixed material, where it would likely have been misinterpreted as slower progression of a sub-population of meiocytes (Valuchova et al., 2020). These examples underscore the power of live-cell imaging.

### Microtubule Rearrangements and Regulation

The cytoskeleton undergoes major rearrangements during meiosis, as illustrated in Arabidopsis. Microtubules are evenly distributed across the cytoplasm at the onset of meiosis but take on an arc-like structure resembling a half-moon during early prophase. Similar to mitosis, a full-moon-like microtubule structure surrounds the nucleus later in prophase; when the nuclear envelope breaks down, microtubules rapidly rearrange to form the first spindle in metaphase I and the second spindle in metaphase II (Prusicki et al., 2019). These different arrangements offer visible native markers for the identification of meiotic stages in the absence of a specific meiotic marker and in an imaging setup that does not reach the same level of chromosomal resolution achieved by cell spreads (Prusicki et al., 2019).

Altered microtubule dynamics can have a strong effect on the meiotic outcome, as seen in the maize mutant *dv1* (*divergent spindle1*), which carries a mutation in *ZmKin6*, a member of the kinesin-14A subfamily of minus end–directed microtubule motor proteins. The *dv1* mutant shows an aberrant spindle shape at metaphase I, ultimately resulting in lagging chromosomes at anaphase I. This phenotype was validated by movies of metaphase I of isolated maize meiocytes (Higgins et al., 2016).

Moreover, microtubules are affected in mutants with lower CDK activity, for example, in *tam*, in which phragmoplast-like structures are observed prior to nuclear envelope breakdown (Prusicki et al., 2019). Similarly, ectopic phragmoplasts were observed when a weak loss-of-function allele of *cdka;1* was combined with mutants of *CDKD;3*, a *CDK-ACTIVATING KINASE* (*cdka;1*^VF^* cdkd;3*). Moreover, meiocytes of *cdka;1*^VF^* cdkd;3* plants also displayed loss of the half-moon and full-moon structures and premature cytokinesis after the first male meiotic division (Sofroni et al., 2020).

### Chromosome Movements and Segregation

Chromosome trajectories during prophase in maize were described by Sheehan and Pawlowski (2009) as following three types of movement: rotational movement of the entire chromatin, rapid short-distance oscillations of extruded chromosome segments, and slower paced movements of chromosome segments located inside the chromatin mass. Moreover, these movements vary considerably between different meiotic phases; zygotene chromosomes move rapidly in a short-range pattern, while chromosome arms move more slowly and cover longer distances in pachytene. Chromosome movements disappear when the anthers are treated with drugs that affect cytoskeleton polymerization (latrunculin B and colchicine), demonstrating that they depend on both actin and tubulin.

The shape of the nuclear envelope (NE) also changes during prophase, not as a consequence of chromosome movements, but due to the force of chromosome movements itself (Sheehan and Pawlowski, 2009). Chromosomes move rapidly in Arabidopsis. In addition, the nucleus and the nucleolus show a characteristic movement pattern during Arabidopsis prophase, with the nucleus moving from a central position during premeiotic stages to a lateral position during early prophase, only to then return to a central position at late pachytene–diplotene. Similarly, the nucleolus is located at the periphery of the nucleus at the onset of leptotene and stays there until diakinesis. Moreover, both the nucleus (chromatin mass) and the nucleolus appear to spin at different speeds during the entire prophase I (Prusicki et al., 2019). The dynamics of prophase chromosomes have been linked to homologous chromosome pairing (Sheehan and Pawlowski, 2009). However, the functional relevance of chromosome movements is not yet fully understood, although it is hoped that live-cell imaging will help reveal their role and regulation.

At later stages of meiosis, chromosomes are highly condensed and are distributed to opposite cell poles during anaphase I and II. These movements are mediated by spindle microtubules attached to kinetochores, multi-protein structures that connect chromosomes to the microtubule fibers of the spindle. The speed of chromosome segregation was accurately measured in maize meiocytes by Yu et al. (1997). Maize chromosomal architecture is characterized by the presence of knobs, also called neocentromeres, which offer a second anchoring point between chromatin and the cytoskeleton besides centromeres or kinetochores. Knobs and kinetochores promoted movements with different kinetics, with speeds of 1.08 and 0.78 mm/min, respectively. However, the faster movements of knobs did not appear to influence the speed of the kinetochore of the same chromosome, ultimately causing a stretching of chromosome arms during anaphase, but not an overall faster chromosome movement, demonstrating that the predominant force during meiotic anaphase was dependent on kinetochore movements (Yu et al., 1997). Furthermore, live-cell imaging revealed that the two pools of homologous chromosomes segregate toward the cell poles following an asymmetric motion: The chromosome mass that is furthest from the edge of the cell moves faster and farther to reestablish the lost symmetry necessary to achieve a balanced cytokinesis. The phragmoplast forms at the half-point between the chromosome masses and not at the spindle zone, as previously hypothesized (Nannas et al., 2016).

### Protein Turnover and Regulation of Meiosis

Live-cell imaging is also a powerful tool to monitor protein abundance and, thus, the regulation of meiotic progression. Examples include the above-mentioned quantitative dissection of CDKA;1 localization (Yang et al., 2020).

Live-cell imaging offered a clear and quantitative picture of the gradual loss of REC8 from chromosomes mediated by WAPL, which provided evidence for the prophase pathway of cohesion removal during meiosis (Yang et al., 2019). Likewise, the molecular mechanisms underpinning the protection of centromeric cohesion at anaphase I were assessed by the imaging of plants expressing *REC8pro:REC8-GFP.* The plant homolog of securin, PANS1, prevents the cleavage of centromeric cohesin by the separase protease. REC8 removal at chromosome arms requires the degradation of PANS1 to release the repression imposed on separase, ultimately promoting the segregation of the chromosome homologs. The direct microscopy observations of REC8-GFP behavior in meiocytes expressing a variant of PANS1 that can no longer be degraded (*DMC1pro:PANS1*Δ*D*) demonstrated that PANS1 degradation is necessary to remove the remaining REC8 from chromosome arms at anaphase I and to promote homolog segregation (Cromer et al., 2019).

## Meiotic Recombination Observations by Live-Cell Imaging

Recombination is a key event during meiosis; therefore, understanding its underlying mechanisms and regulatory principles is important in many fundamental and applied aspects of plant life: to obtain insights into genome evolution, biodiversity, and breeding (Grelon, 2016; Lambing et al., 2017; Wang and Copenhaver, 2018).

Several techniques have been adopted to investigate recombination, ranging from immunolocalization on cell spreads and *in situ* hybridization (among many: Schwarzacher, 2003; Kurzbauer et al., 2018; Martinez-Garcia et al., 2018; Sims et al., 2019) to three-dimensional immunolocalization (Hurel et al., 2018), fluorescent transgenic lines (FTLs) to visualize segregation (Francis et al., 2007), and genotyping by sequencing or chromatin immunoprecipitation followed by sequencing (ChIP-seq) methods to explore the role of epigenetic modifications on recombination cold and hot spots (Pawlowski et al., 2013; Lambing and Heckmann, 2018; Pradillo and Heckmann, 2020).

Being able to follow recombination in real time will open the door to a new level of understanding of this central aspect of meiosis. A first example comes from live-cell imaging of meiosis in Arabidopsis under heat stress (34°C) that uncovered the presence of a long-doubted recombination checkpoint, also known as pachytene checkpoint (De Jaeger-Braet et al., 2021). In animals and yeast, aberrant recombination structures and the complete loss of recombination cause a delay of pachytene (Bishop et al., 1992; Rockmill et al., 1995; Barchi et al., 2005). This arrest is controlled by the checkpoint kinase ATAXIA TELANGIECTASIA MUTATED (ATM). Since in Arabidopsis and other plants the loss of recombination, as for instance seen in *dmc1* mutants, does not cause meiotic arrest, it was concluded that plants do not possess a pachytene checkpoint (Couteau et al., 1999; Grelon et al., 2001; Caryl et al., 2003; Higgins et al., 2004; Li et al., 2004, 2009; Jones and Franklin, 2008; Wijnker and Schnittger, 2013). However, imaging *dmc1* and other mutants in which recombination is abolished such as *spo11-1* at 34°C revealed that the duration of pachytene reverted to almost the level seen in the wildtype at 21°C indicating that the pachytene delay is recombination dependent. Importantly, the pachytene delay in Arabidopsis is also absent in *atm* mutants exposed to 34°C (De Jaeger-Braet et al., 2021). Thus, it appears that there is a pachytene checkpoint in plants. However, this checkpoint likely monitors aberrant recombination structures rather than the absence of COs.

Now, it will be very interesting to further zoom into these defective recombination structures knowing that heat stress and other environmental conditions can modify the position and number of COs (Phillips et al., 2015; Morgan et al., 2017; De Storme and Geelen, 2020). To this end, it will be very informative to follow the recombination machinery itself. However, live-cell imaging of recombination is challenging and is largely limited by three important factors: the current achievable resolution, the movement of chromosomes, and the availability of reporter lines to label proteins of the recombination machinery and to highlight specific chromosomal regions such as the 45S rDNA region and telomeres *in vivo*.

As outlined above, the dynamics of the meiotic chromosome axis, which is key for recombination, is well established by following components such as ASY1, ASY3, and REC8. New reporter lines and live-cell imaging assays now need to be established for the analysis of DSB formation, strand invasion, and CO resolution. These new tools will make it possible to study the appearance and disappearance of each component of the recombination machinery to determine their relative order and how the recombination machinery is affected in various mutant backgrounds.

These studies may explain how a defined DSB site is resolved as a CO, how a CO is assigned to class I or class II, or how CO interference is established, which is far from being fully understood. As recently shown by immuno-cytochemistry, the E3 ligase HEI10, which is a crucial determinant of type I COs and CO interference, is present along the chromosome axis in early pachytene. It then accumulates in growing foci, which are evenly distributed at the expense of smaller, closely spaced peaks in mid and late pachytene (Morgan et al., 2021). It will be now very interesting to follow these foci in real time and correlate their dynamics with other type I CO components by live-cell imaging.

This work may also be extendable to studying recombination during female meiosis, which is differently regulated in both Arabidopsis and crop species (Giraut et al., 2011; Qin et al., 2014; Zhao et al., 2014; Kianian et al., 2018). Furthermore, live-cell imaging has also the great potential to contribute to an understanding of species-specific difference in meiosis. For instance, the duration of leptotene and early pachytene was extended in *spo11-1* mutants in Arabidopsis while in yeast, the corresponding mutants progress faster through meiosis than the wildtype (Klapholz et al., 1985; Jiao et al., 1999; Cha et al., 2000; De Jaeger-Braet et al., 2021). Thus, the era of live-cell imaging for meiosis has just begun, with many exciting discoveries ahead of us.

## Author Contributions

MP, MB, KS, YH, and AS selected the content and the references, contributed to define the content structure and revised and approved the article. MP, MB and KS designed the figures and the tables. MP, MB and AS wrote the article. All authors contributed to the article and approved the submitted version.

## Conflict of Interest

The authors declare that the research was conducted in the absence of any commercial or financial relationships that could be construed as a potential conflict of interest.

## Publisher’s Note

All claims expressed in this article are solely those of the authors and do not necessarily represent those of their affiliated organizations, or those of the publisher, the editors and the reviewers. Any product that may be evaluated in this article, or claim that may be made by its manufacturer, is not guaranteed or endorsed by the publisher.

## Footnotes

1
https://t1p.de/ypjk
